# Functional Analysis of the P-Type ATPases Apt2-4 from *Cryptococcus neoformans* by Heterologous Expression in *Saccharomyces cerevisiae*

**DOI:** 10.3390/jof9020202

**Published:** 2023-02-04

**Authors:** Sarina Veit, Sabine Laerbusch, Rosa L. López-Marqués, Thomas Günther Pomorski

**Affiliations:** 1 Department of Molecular Biochemistry, Faculty of Chemistry and Biochemistry, Ruhr University Bochum, 44801 Bochum, Germany; 2 Department of Plant and Environmental Sciences, University of Copenhagen, Thorvaldsensvej 40, DK-1871 Frederiksberg C, Denmark

**Keywords:** β-subunit, CDC50 protein, functional complementation, heterologous expression, lipid transport, lipid flippase, P-type ATPase

## Abstract

Lipid flippases of the P4-ATPase family actively transport phospholipids across cell membranes, an activity essential for key cellular processes such as vesicle budding and membrane trafficking. Members of this transporter family have also been implicated in the development of drug resistance in fungi. The encapsulated fungal pathogen *Cryptococcus neoformans* contains four P4-ATPases, among which Apt2-4p are poorly characterized. Using heterologous expression in the flippase-deficient *S. cerevisiae* strain *dnf1Δdnf2Δdrs2Δ*, we tested their lipid flippase activity in comparison to Apt1p using complementation tests and fluorescent lipid uptake assays. Apt2p and Apt3p required the co-expression of the *C. neoformans* Cdc50 protein for activity. Apt2p/Cdc50p displayed a narrow substrate specificity, limited to phosphatidylethanolamine and –choline. Despite its inability to transport fluorescent lipids, the Apt3p/Cdc50p complex still rescued the cold-sensitive phenotype of *dnf1Δdnf2Δdrs2Δ*, suggesting a functional role for the flippase in the secretory pathway. Apt4p, the closest homolog to Saccharomyces Neo1p, which does not require a Cdc50 protein, was unable to complement several flippase-deficient mutant phenotypes, neither in the presence nor absence of a β-subunit. These results identify *C. neoformans* Cdc50 as an essential subunit for Apt1-3p and provide a first insight into the molecular mechanisms underlying their physiological functions.

## 1. Introduction

Cryptococcosis is a severe infection of the central nervous system, especially the brain tissue, that leads to meningoencephalitis and is the second most common cause of death in immunocompromised HIV patients [1]. It is predominantly caused by the encapsulated yeast *Cryptococcus neoformans*, which enters the blood stream through the lung, thereby occasionally resulting in additional pneumonia [2]. In 2022, the World Helath Organization (WHO) declared this as a critical pathogen for which research and development efforts should be prioritized [3]. The currently available drugs against *C. neoformans* are limited by their ability to cross the blood-brain barrier and their toxicity causes severe side effects. In addition, the requirement for prolonged treatment (6–12 months) favors the development of fungal drug resistance [4,5]. Novel therapeutic strategies could target the capsule morphology and polysaccharide secretion, which are crucial for the virulence of *C. neoformans*, highlighting the role that the secretory pathway plays in its pathogenicity [6].

Throughout secretion and especially for vesicular transport, cellular membranes undergo dynamic morphological changes and re-organization in terms of lipid composition and distribution over the two leaflets of the bilayer. A key player in these processes are P4-ATPases that catalyze the translocation of phospholipids from the exoplasmic to the cytosolic leaflet of biological membranes, a process termed “lipid flipping” [7]. Members of this transporter family have also been implicated in the development of drug resistance in fungi, which includes resistance to alkyllysophospholipids [8,9,10]. Due to their importance in fungal infection, the functional characterization of these so-called flippases is essential to understanding the infection mechanism and subsequently using them as new fungal drug targets, as recently shown for the malaria parasite [11].

In fungi, the family of P4-ATPases is best studied for *S. cerevisiae*. This non-pathogenic yeast expresses five P4-ATPases, including Dnf1p and Dnf2p at the plasma membrane (PM), Drs2p and Dnf3p mostly in the trans-Golgi network, and Neo1p in the endosomal membranes and Golgi [12,13]. In *C. neoformans*, only four P4-ATPases, Apt1-4p, were identified by sequence alignment [14,15]. Deletion studies in vivo revealed that Apt1p plays an important role in polysaccharide export, capsule size, and virulence [14,16]. In contrast, separate *APT2-4* deletions did not show any detectable effect except for the *APT3* deletion, which caused a minor increase in sensitivity to the trafficking inhibitor BFA, as well as fluconazole, an inhibitor for ergosterol biosynthesis [17]. However, functional redundancy among flippases has been observed before, emphasizing the importance of heterologous expression strategies for proper analysis [18]. In fact, Apt1p was recently functionally characterized based on heterologous expression in *S. cerevisiae* as a lipid transporter with broad substrate specificity. Besides the glycerophospholipids phosphatidylserine (PS), -choline (PC), -ethanolamine (PE), and -glycerol (PG), the transport of galactosyl ceramide (GalCer), glucosyl ceramide (GlcCer), and sphingomyelin (SM) was detected [19]. Substrate specificity and key features of Apt2-4p remain to be assessed.

Most P4-ATPases are functional only as a heterodimer with their β-subunit, which is involved in the localization to the destination membrane, as well as in the lipid transport activity itself [8,20,21,22]. While the *S. cerevisiae* genome contains three genes coding for β-subunits (*CDC50*, *LEM3*, and *CRF1*) [23], only one gene of this family, previously named *CDC50*, was identified in the genome of *C. neoformans*. Studies in vivo demonstrated that Cdc50p localizes throughout the whole secretory pathway and its deletion leads to reduced virulence, higher drug sensitivity, and exposure of phosphatidylserine on the outer leaflet of the plasma membrane [9,17]. Further studies showed impaired lipid uptake at the plasma membrane via Apt1p, when expressed without Cdc50p, presumably due to impaired trafficking and therefore the retention of Apt1p in the endoplasmic reticulum [19]. Moreover, recent studies showed a strong interaction between subunits via the extracellular loop of Cdc50p, which motivated the design of antifungal peptides [24]. The subclass of P4B-ATPases seems to work without a β-subunit and comprises among others Neo1p from *S. cerevisiae*, which shares high sequence similarities with Apt4p [25].

In this study, we performed a functional characterization of Apt2p, Apt3p, and Apt4p in comparison to Apt1p through heterologous expression. We employed a well-established system based on the *dnf1Δdnf2Δdrs2Δ* background in *S. cerevisiae* [26]. This mutant strain is deleted in three out of five endogenous P4-ATPases, consequently displaying a low background of phospholipid uptake at the plasma membrane and altered membrane lipid asymmetry. Since previous studies did not show clear phenotypes for single *APT2-4* deletion mutants due to redundancy, we used the heterologous expression system to provide first insight into the subcellular localization and substrate specificity of Apt2-4p, and to help unravel their physiological functions. Furthermore, we addressed the dependency on Cdc50p co-expression for proper Apt protein localization and flippase activity.

## 2. Materials and Methods

### 2.1. Materials

Miltefosine (#850337) and most NBD-lipids were purchased from Avanti Polar Lipids (Alabaster, AL, USA), including 1-palmitoyl-2-{6-[(7-nitro-2-1,3-benzoxadiazol-4-yl)amino]hexanoyl}-sn-glycero-3-phosphocholine (NBD-PC; #810130), 1-palmitoyl-2-{6-[(7-nitro-2-1,3-benzoxadiazol-4-yl)amino]hexanoyl}-sn-glycero-3-phosphoethanol-amine (NBD-PE; #810153), 1-palmitoyl-2-{6-[(7-nitro-2-1,3-benzoxadiazol-4-yl)amino]hexanoyl}-sn-glycero-3-phosphoserine (ammonium salt) (NBD-PS; #810192), 1-palmitoyl-2-{6-[(7-nitro-2-1,3-benzoxadiazol-4-yl)amino]hexanoyl}-sn-glycero-3-[phospho-rac-(1-glycerol)] (ammonium salt) (NBD-PG; #810163), N-[6-[(7-nitro-2-1,3-benzoxadiazol-4-yl)amino]hexanoyl]-sphingosine-1-phosphocholine (NBD-SM; #810218), N-[6-[(7-nitro-2-1,3-benzoxadiazol-4-yl)amino]hexanoyl]-D-galactosyl-β1-1′-sphingosine (NBD-GalCer; #810220), and N-[6-[(7-nitro-2-1,3-benzoxadiazol-4-yl)amino]hexanoyl]-D-glucosyl-β1-1′-sphingosine (NBD-GlcCer; #810222). N-[(1S,2R,3E)-1-[[(4-O-beta-D-galactopyranosyl-beta-D-glucopyranosyl)oxy]methyl]-2-hydroxy-3-heptadecen-1-yl]-hexadecanamide-d3 (NBD-LacCer, #Cay24625-1) was purchased from Biomol (Hamburg, Germany). The detergent n-dodecyl-β-D-maltopyranoside (DDM) was purchased from GlyconBiochemicals GmbH (Luckenwalde, Germany). Unless otherwise indicated, chemicals were obtained from Sigma-Aldrich (München, Germany). Protease inhibitor cocktail contained aprotinin (1 mg/mL), leupeptin (1 mg/mL), pepstatin A (1 mg/mL; Roth), antipain (5 mg/mL), and benzamidine (0.157 mg/mL) in dimethylsulfoxide and was used at a 1:1000 dilution.

### 2.2. Plasmid Construction

Primers and plasmids used in this study are listed in Supplementary Tables S1 and S2, respectively. Stellar^TM^ Competent Cells included in the InFusion cloning kit (Takara Clontech, Mountain View, CA, USA) were used for this work following manufacturer’s instructions. Transformed cells were grown in LB medium (0.5% (*w*/*v*) yeast extract, 1% (*w*/*v*) peptone, 1% (*w*/*v*) NaCl, 1.5% (*w*/*v*) agar for plates) supplemented with 100 µg/mL ampicillin. Standard PCR reactions were performed with Q5 High-Fidelity DNA Polymerase (NEB, Ipswich, MA, USA). The pESC-URA vector (Agilent Technologies, Santa Clara, CA, USA) digested with BamHI was used as the backbone for *APT*-containing constructs without *CDC50*, while the pESC-URA-APT1-GFP-CDC50-FLAG plasmid [19] digested with the same enzyme was utilized for *CDC50*-containing versions. The *APT2*, *APT3*, and *APT4* sequences were obtained as synthetic cDNA clones from Twist Bioscience (San Francisco, CA, USA). *APT3* and *APT4* were purchased as full length sequences (APT3_J9VGP8; APT4_J9VM87) and *APT2* as two fragments (APT2_J9VQH2_Nt, APT2_J9VQH2_Ct). *APT2* C- and N-terminal fragments were amplified with primer sets #2-Nt and #2-Ct, respectively. As a template for the myc-tagged fragment with a GSGSGSG linker, the pESC-URA-APT1-myc-CDC50-FLAG [19] was used and amplified with the respective primer set (#2-myc). The three overlapping fragments were introduced into the corresponding vectors by InFusion cloning (Takara Clontech) yielding pESC-URA-APT2-myc-CDC50-FLAG and pESC-URA-APT2-myc. Likewise, *APT3* and *APT4* were amplified with primer sets #3 and #4, and their corresponding myc fragment using primer sets #3-myc and #4-myc. After InFusion cloning into both vectors, pESC-URA-APT3-myc-CDC50-FLAG, pESC-URA-APT3-myc, pESC-URA-APT4-myc-CDC50-FLAG, and pESC-URA-APT4-myc were obtained. Similarly, pESC-URA-APT1-GFP-CDC50-FLAG was used as a template for GFP-versions with a GSGSGSG linker, using primer sets #2-GFP, #3-GFP and #4-GFP. Infusion cloning with the corresponding *APT2*, *APT3* and *APT4* fragments into both vectors yielded pESC-URA-APT2-GFP-CDC50-FLAG, pESC-URA-APT2-GFP, pESC-URA-APT3-GFP-CDC50-FLAG, pESC-URA-APT3-GFP, pESC-URA-APT4-GFP-CDC50-FLAG, and pESC-URA-APT4-GFP. All constructs were verified by sequencing.

### 2.3. Yeast Strains and Media

The flippase-deficient strain *dnf1Δdnf2Δdrs2Δ* (ZHY709; MAT*α his3 leu2 ura3 met15 dnf1∆ dnf2∆ drs2*::*LEU2*; [26]) and corresponding wild-type strain BY4741 (MAT*a his3 leu2 ura3 met15*; EUROSCARF), and MTY628-15BL (MAT*a his3_1 leu2_0 ura3_0 lys2_0 neo1_::KanMX* pRS315-*neo1-1*; [25]) with MTY219RRL (MAT*a his3_1 leu2_0 ura3_0 lys2_0 neo1_::KanMX* pRS315-*NEO1*; [25]) as wild-type strain were transformed fresh for every experiment using the lithium acetate method [27] and cultured at 28 °C in selective standard synthetic glucose (SD) or galactose (SG) medium containing 0.7% (*w*/*v*) Yeast Nitrogen base without amino acids (Formedium, Hunstanton, UK), 0.192% (*w*/*v*) drop-out mix without uracil and 2% (*w*/*v*) glucose or galactose, respectively. For solid medium, 2% (*w*/*v*) agar was added.

### 2.4. Functional Complementation Assays

For growth tests, cells were cultivated overnight in liquid SD media at 28 °C with 100 rpm shaking and diluted with ddH_2_O to an OD_600_ of 0.1 before drops of 3 µL were spotted on SG plates and incubated at 28 °C for 4 days. For gradient plates containing cytotoxic drugs, a maximum of 0.75 µM duramycin and 144 µg/mL miltefosine was used. For cold-sensitivity tests, serial 1:5 dilutions with starting OD_600_ of 0.2 were spotted on SG and SD plates and incubated at 20 °C for ten days. In the case of *NEO1* complementation tests, cells were spotted as serial 1:5 dilutions with starting OD_600_ 0.3 on Yeast Peptone Galactose (YPG) plates containing 2% (*w*/*v*) peptone, 1% (*w*/*v*) yeast extract, 2% (*w*/*v*) galactose and 2% (*w*/*v*) agar and were incubated either at 30 °C (control) or above the restrictive temperature of the *neo1-1^ts^* strain at 35 °C [13,25] for 48 h.

### 2.5. Membrane Preparation and Gradient Fractionation

Fresh transformants (3 to 10 colonies) were used to inoculate 100 mL of selective SD medium followed by incubation (16 h, 28 °C, 100 rpm). Cells were washed and 500 mL of selective SG medium was inoculated with a start OD_600_ of 0.5. After expression at 25 °C and 100 rpm for 26 h, cells were harvested, washed, and lysed mechanically with the use of 0.5 mm acid-washed glass beads in ice-cold lysis buffer I (0.8 M sorbitol, 10 mM EDTA, 50 mM HEPES-KOH, pH 7.2) supplemented with protease inhibitor cocktail and 0.25 mM phenylmethylsulfonyl fluoride (PMSF). The lysate was cleared by centrifugation (10 min, 500× *g*, 4 °C), and membranes were collected from the supernatant by further centrifugation (130,000× *g*, 1 h, 4 °C). For fractionation studies, membranes corresponding to an OD_600_ of 1 in 1 L of culture were loaded on top of a sucrose gradient (from bottom to top, 1.25 mL of 53, 43, 33.5 and 29.2% (*w*/*w*) sucrose prepared in lysis buffer I) and centrifuged in a swinging buckets rotor (SW32 Ti) at 130,000× *g* for 17 h at 4 °C. Fractions (500 µL) were collected from the top, diluted with lysis buffer I, and membranes were collected from each fraction by centrifugation (130,000× *g*, 1 h, 4 °C) followed by immunoblotting. Protein blots were probed with anti-Pma1p antibodies (Invitrogen, Waltham, MA, USA; #MA1-91567) to detect the plasma membrane and anti-Dpm1p antibodies (Invitrogen; #A6429) for the endoplasmic reticulum, respectively. Apt-myc proteins were detected via anti-myc antibodies (Invitrogen; #13-2500) and Cdc50-FLAG was detected via anti-FLAG M2 antibody (Sigma-Aldrich; #F3165).

### 2.6. Co-Immunoprecipitation

For co-immunoprecipitation, lysis buffer II (150 mM NaCl, 20 mM HEPES-NaOH, pH 7.4) was used and the procedure was performed as described previously [19]. Briefly, membranes were solubilized with 0.6% (*w*/*v*) n-dodecyl-β-D-maltopiranoside (DDM) at a protein concentration of 1 mg/mL. The solubilized fraction was loaded on an anti-FLAG M2 matrix and incubated for 16 h with end-over-end rotation, followed by elution with FLAG-peptide. During the purification steps, samples were collected and analyzed by immunoblotting.

### 2.7. NBD-lipid Uptake and Flow Cytometry

Fresh transformants (3 to 10 colonies) were inoculated in 50 mL of selective SG medium. Cultures were incubated in Erlenmeyer flasks at 25 °C and 110 rpm for 16 h to induce expression. After dilution to an OD_600_ of 0.2 in a total volume of 50 mL, cultures were incubated for another 4 h under the same conditions to ensure similar growth. Afterwards, the NBD-lipid uptake was performed as described in [28]. Briefly, cells were washed and resuspended in selective SG media to an OD_600_ of 8. Subsequently, 250 µL of cells were transferred to a conical glass tube and incubated in a 30 °C water bath for 10 min. Short-chain NBD-lipids were dissolved in dimethylsulfoxide (DMSO) to a concentration of 10 mM and 1.5 µL of the solution were added to the cells. After incubation at 30 °C for 30 min, the transport was terminated by placing the cells on ice and addition of sodium azide. Subsequently, excess NBD-lipids were extracted with bovine serum albumin (BSA) and, finally, cells were resuspended in 250 µL phosphate buffered saline (PBS; 150 mM NaCl, 8 mM Na_2_HPO_4_, 2 mM KH_2_PO_4_, pH 7.0). For flow cytometric analysis, 50 µL of cell suspension were labeled with 1 µg propidium iodide, diluted with 1 mL PBS and analyzed with a CyFlow^®^ SL cytometer (Sysmex Partec, Goerlitz, Germany). Using a flow rate of 600 cells/s, a total of 20,000 cells were measured. Viable cells were selected based on forward/side-scatter gating and propidium iodide exclusion. Data were analyzed with FlowJo^TM^ (BD Life Sciences-FlowJo, Ashland, OR, USA).

### 2.8. Sequence Alignment and Similarity Analysis

Protein sequences and structures were obtained from Uniprot and PDB, respectively. From *C. neoformans*: Apt1p (J9VZ19), Apt2p (J9VQH2), Apt3p (J9VGP8), Apt4p (J9VM87), Cdc50p (J9VW44); from *S. cerevisiae*: Dnf1p (P32660; 7kyc), Dnf2p (Q12675; 7kya), Dnf3p (Q12674), Drs2p (P39524; 6roh), Neo1p (P40527; 7rd7). Sequence alignment for phylogenetic analysis was performed in MEGAX [29] with MUSCLE [30]. Identity and similarity values are based on EMBOSS Needle online tool for pairwise sequence alignment [31]. The alignment was further used, together with PDB structures for corresponding *S. cerevisiae* homologs, as target-template to predict the transmembrane domains of Apt1-4p using SWISS-MODEL [32]. Pfam [33] was used to identify protein family domains and topology was visualized with Protter [34]. Glycosylation sites in Cdc50p were predicted using the NetGlyc server [35].

## 3. Results

### 3.1. Expression of Heterologous APT1-4 and CDC50 in S. cerevisiae

**Figure 1:**
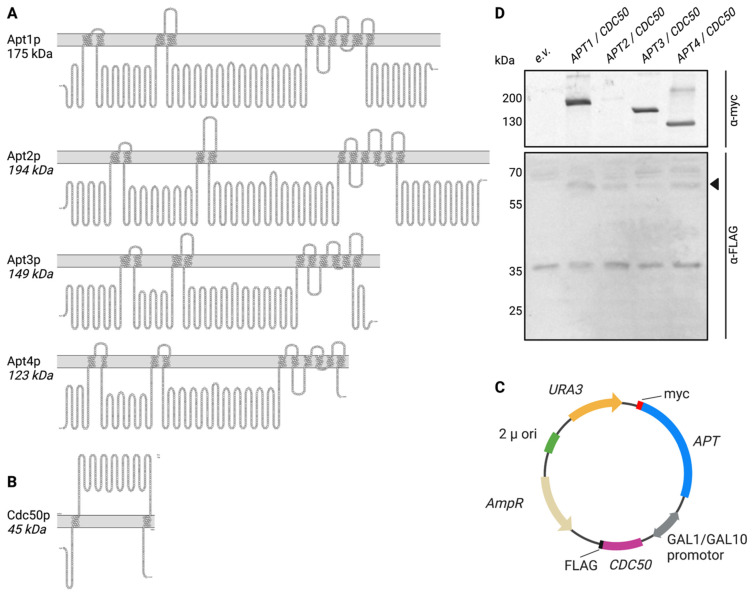
Topology and heterologous expression of *APT1-4/CDC50* in the P4-ATPase-deficient S. cerevisiae strain *dnf1Δdnf2Δdrs2Δ*. Topology prediction of Apt1-4p (**A**) Cdc50p (**B**) based on sequence alignment with corresponding *S. cerevisiae* homologs was visualized with Protter. (**C**): architecture of pESC-URA-based plasmids used to express myc-tagged *APT1-4* together with FLAG-tagged *CDC50* under a bidirectional GAL1/GAL10-promoter. *2µ ori*: origin of replication; *AmpR*: ampicillin resistance gene, *URA3*: uracil selection marker. (**D**): Immunoblot showing the expression of myc-tagged Apt1-4p and FLAG-tagged Cdc50p (black arrowhead) in the P4-ATPase-deficient *S. cerevisiae* strain *dnf1Δdnf2Δdrs2Δ* in comparison to the empty vector (e.v.). The mobilities of marker proteins of known mass (kDa) are indicated on the left.

Sequence homology searches revealed that, in addition to the previously studied Apt1p [19] three putative P4-ATPases Apt2p, Apt3p, and Apt4p are present in *C. neoformans* [19,36]. Apt2p, Apt3p, and Apt4p contain all the features of functional P-type ATPases, including the presence of 10 hydrophobic membrane-spanning helices (Figure 1A) and a characteristic pattern of conserved residues (Supplementary Figure S1). Common motifs found in all P4-ATPases [37] include: (i) the DKTGT motif, containing an aspartate that undergoes phosphorylation–dephosphorylation cycles during ATP hydrolysis and is typical for all P-type ATPases; (ii) a DGET motif that acts as an in-built phosphatase and enables dephosphorylation of the aspartate residue in the DKTGT motif; (iii) Conserved motifs TGD(K/R) and GDG(A/G)ND in the big cytosolic loop between transmembrane helix (TM)4 and TM5 necessary for Mg^2+^ binding; (iv) a conserved PISL motif in TM4 that is essential for lipid flipping. In agreement to its similarity to P4B-ATPase, Apt4p contains a IHRG motif in TM5 (FYNK in P4A-ATPases) and a small deletion of 6-8 amino acids after TM4 with respect to Apt1-3p [38,39].

To facilitate functional studies of Apt1-4p, the corresponding genes were cloned in a pESC-URA vector containing a bidirectional galactose-inducible GAL1/GAL10 promoter for expression in the flippase-deficient yeast strain *dnf1Δdnf2Δdrs2Δ*. Apt1-4p were C-terminally tagged with a myc-tag while an N-terminal FLAG-tag was added to Cdc50p (Figure 1C). After heterologous expression at 25 °C to facilitate proper folding, total membrane extracts were analyzed by immunoblotting. As shown in Figure 1D, all *APT*s are expressed, as demonstrated by the detection of bands of the predicted size (Figure 1A), albeit Apt2p shows a significantly lower expression level than the other Apt proteins. Cdc50p is expressed to comparable levels in all strains and detected as a band of higher molecular weight than expected based on the amino acid sequence, due to glycosylation [19].

### 3.2. Subcellular Localization of Heterologous Apt1-4p and Interaction with Cdc50p

**Figure 2:**
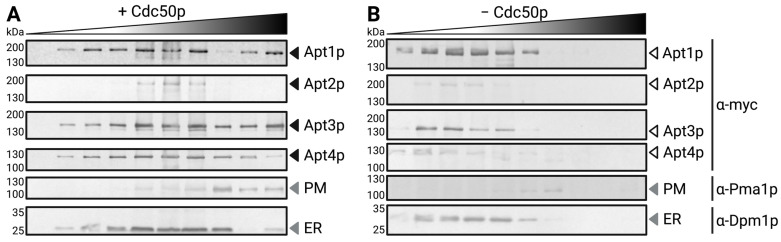
Intracellular localization of Apt proteins upon heterologous expression in the P4-ATPase-deficient *S. cerevisiae* strain *dnf1Δdnf2Δdrs2Δ*. (**A**,**B**): *dnf1Δdnf2Δdrs2Δ* yeast cells expressing myc-tagged Apt1-4p with ((**A**), black arrows) or without ((**B**), white arrows) FLAG-tagged Cdc50p were lysed, and cellular membranes were fractionated on a sucrose step gradient. Gradient fractions were immunoblotted using antibodies against the myc-epitope or several organellar markers. Fractionation profiles of protein markers correspond to cells expressing myc-tagged *APT3* but were determined for each gradient individually with similar results. PM, plasma membrane; ER, endoplasmic reticulum.

For determination of Apt1-4p subcellular localization and its dependency on the presence of Cdc50p, total membranes were fractionated by a discontinuous sucrose gradient (Figure 2). In co-expression with Cdc50p, Apt1p and Apt3p clearly reached the plasma membrane. In contrast, only a small amount of Apt4p was detectable in the plasma membrane fraction, while Apt2p localized to intracellular membranes. In the absence of Cdc50p, all four P4-ATPases displayed a prominent intracellular localization. Results were confirmed by heterologous expression of GFP-tagged Apt variants followed by fluorescence microscopy (Supplementary Figure S2).

**Figure 3:**
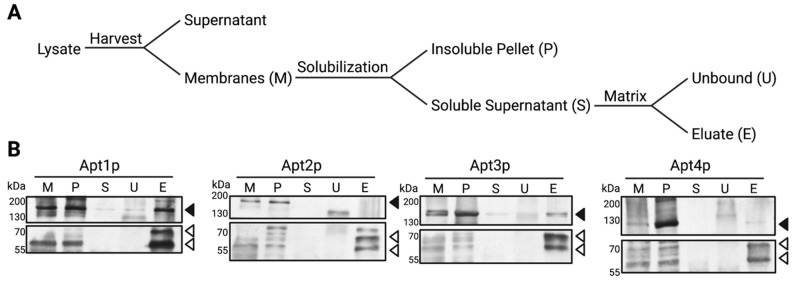
Co-immunoprecipitation of Apt proteins with Cdc50p-FLAG. (**A**): FLAG-affinity purification scheme. After cell lysis, membranes were harvested by centrifugation and solubilized with 0.6% (*w/v*) DDM. Insoluble material was pelleted by ultracentrifugation and the soluble supernatant was incubated with FLAG-affinity matrix to bind Cdc50p-FLAG. The supernatant of this incubation containing unbound proteins was discarded and bound proteins were eluted by FLAG-peptide addition. (**B**): Apt1-4p-myc were expressed with Cdc50p-FLAG and purified according to (**A**). Equal volumes of each fraction were loaded onto SDS-PAGE gels, except for the eluate fraction, for which a 10× volume was loaded. Apt1-4p and Cdc50p were detected via anti-myc (black arrows) and anti-FLAG antibodies (white arrows), respectively. Glycosylation variants are indicated for Cdc50p.

To test for a physical interaction between Apt1-4p and Cdc50p, co-immunoprecipitation by FLAG-purification was performed (Figure 3A). Total membranes from cells co-expressing Cdc50p-FLAG with the respective Apt proteins were solubilized with detergent and subjected to anti-FLAG-affinity purification. Unbound non-interacting proteins were then discarded and bound proteins were eluted using a buffer containing FLAG-peptide. The fractions collected during affinity purification were subsequently analyzed via immunoblot (Figure 3B). Apt1p and Apt3p were detectable in the eluates, indicating a strong interaction with Cdc50p-FLAG. However, we were unable to solubilize Apt2p and Apt4p, preventing any conclusions about their interaction with the β-subunit by this approach.

### 3.3. Functional Status of Heterologously Expressed Apt1-4p in S. cerevisiae

**Figure 4:**
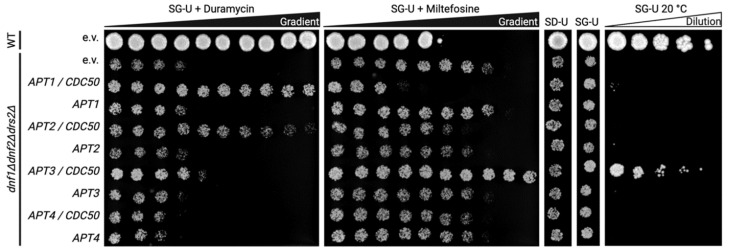
Complementation analysis for *APT1-4/CDC50* combinations expressed in the P4-ATPase-deficient *S. cerevisiae* strain *dnf1Δdnf2Δdrs2Δ*. Functional complementation of the P4-ATPase-deficient yeast strain *dnf1Δdnf2Δdrs2Δ* expressing myc-tagged Apt1-4p with and without FLAG-tagged Cdc50p. Wild-type (BY4741; WT) and triple mutant cells transformed with empty plasmids were used as positive and negative controls, respectively. Cells were spotted at a constant 0.1 OD_600_ on glucose- (SD, control) or galactose-containing (SG, induced gene expression) plates supplemented with a concentration gradient of the indicated toxins (the direction of the gradient is indicated by a filled triangle) and incubated at 28 °C for three days. For cold-sensitivity testing, serial 1:5 dilutions starting at 0.2 OD_600_ were spotted on SG plates and incubated at 20 °C for ten days. The experiments were repeated three times with identical results.

For functional analysis, flippase-deficient *dnf1Δdnf2Δdrs2Δ* cells expressing *APT1-4* genes either alone or in combination with *CDC50* were spotted on different selective plates (Figure 4). The flippase-deficient strain *dnf1Δdnf2Δdrs2Δ* or the corresponding wild-type strain (BY4741) transformed with the empty vector were used as negative and positive controls, respectively. Cytotoxic drugs were added to the plates to probe for lipid translocation. Duramycin is a small pore-forming tetracyclic peptide that binds to exposed PE at the cell surface and causes cell death [40,41]. In the presence of increasing concentrations of duramycin, *dnf1Δdnf2Δdrs2Δ* cells failed to grow due to their flippase deficiency. However, growth was recovered upon co-expression of *CDC50* with either *APT1*, *APT2* or *APT3*, albeit to different extents, implying a PE flippase activity of the P4-ATPases strictly in complex with the β-subunit. *APT4* failed to complement the *dnf1Δdnf2Δdrs2Δ* phenotype. Miltefosine is a choline-containing lyso-PC analog that is toxic only after uptake across the plasma membrane [5]. Wild-type yeasts are sensitive to miltefosine, whereas *dnf1Δdnf2Δdrs2Δ* is not due to the lack of plasma-membrane-localized flippases (Figure 4). Upon co-expression of *CDC50* with either *APT1* or *APT2*, cells became sensitized to miltefosine, whereas cells co-expressing *CDC50* and *APT4* remained tolerant. Interestingly, the *APT3* transformants showed an even lower sensitivity for miltefosine than the negative empty vector control. This *CDC50*-dependent increase in cell viability is also reflected in the cell density per drop throughout all galactose plates. Furthermore, the *dnf1Δdnf2Δdrs2Δ* cells display a cold-sensitive phenotype [26] that was rescued by co-expression of *APT3* and *CDC50*, but not by any other *APT/CDC50* combination, indicating an intracellular (flippase) activity.

**Figure 5:**
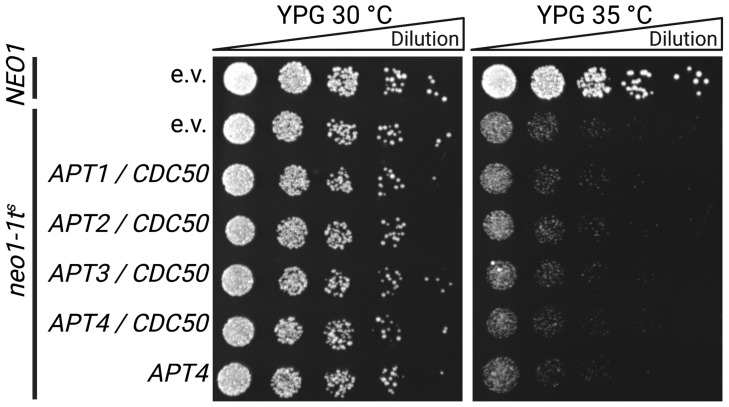
*neo1-1^ts^* complementation assay. A *neo1* deletion strain expressing a temperature-sensitive allele of *NEO1 (neo1-1^ts^)* was transformed with an empty vector (e.v., negative control) or with plasmids bearing *APT-myc* genes and *CDC50-FLAG*. A *neo1* deletion strain expressing *NEO1* from a plasmid was transformed with an e.v. as positive control. Serial 1:5 dilutions starting at 0.3 OD_600_ (indicated by triangles) were spotted on rich galactose plates to induce expression and incubated at 30 °C (control) or above the *neo1-1^ts^* restrictive temperature at 35 °C for 48 h. The experiment was repeated three times with identical results.

*S. cerevisiae* strains lacking *NEO1* are not viable, but deletion of the gene is possible when the yeast is simultaneously complemented with a plasmid-borne temperature-sensitive allele of *NEO1*, as is the case for the *neo1-1^ts^* strain [25]. Above the restrictive temperature of 34 °C, the mutant *neo1-1^ts^* protein is inactive, and cell growth can only be recovered upon expression of an additional active, non-temperature-sensitive version of Neo1p. The *neo1-1^ts^* strain was transformed with the empty vector or with *APT1-4* together with *CDC50* and dilutions were spotted on YPG plates followed by incubation at 30 °C or 35 °C (Figure 5). As a positive control, a *Δneo1* strain complemented with a plasmid bearing wild-type *NEO1* was used. Apt1-4p/Cdc50p complexes were unable to complement the *NEO1* deficiency of *neo1-1^ts^* cells, which grew similarly to the empty vector control. As Apt4p is the closest homolog of Neo1p, which does not require a β-subunit, we also tested Apt4p in the absence of Cdc50p co-expression and observed no functional complementation.

### 3.4. Heterologously Expressed Apt1p and Apt2p Support Lipid Flippase Activity

**Figure 6:**
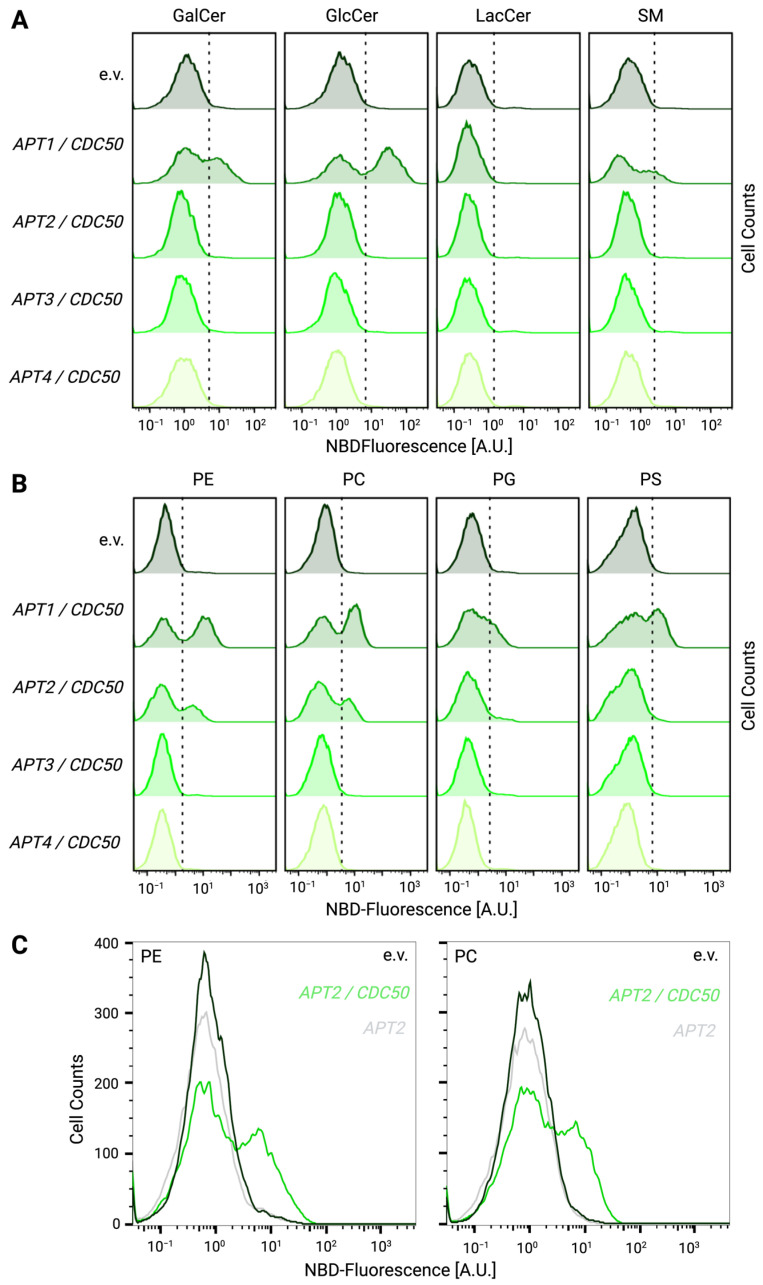
Lipid flippase activity of Apt1-4p/Cdc50p upon heterologous expression in the P4-ATPase-deficient *S. cerevisiae dnf1Δdnf2Δdrs2Δ* strain. *dnf1Δdnf2Δdrs2Δ* yeast cells expressing *APT1-4/CDC50* were labeled with the indicated NBD-lipids and analyzed by flow cytometry. At least 10,000 cells are represented by each histogram. Cells that present higher fluorescence than the threshold (dotted vertical line) were considered as cells positive for flippase activity. (**A**): C6-NBD-sphingolipids tested included Galactosyl ceramide (GalCer), Glucosyl ceramide GlcCer), Lactosyl ceramide (LacCer), and Sphingomyelin (SM). (**B**): C6-NBD-glycerophospholipids tested included Phosphatidylcholine (PC), Phosphatidylethanolamine (PE), Phosphatidylglycerol (PG), Phosphatidylserine (PS), (**C**): *APT2* was analyzed with and without *CDC50* co-expression for PE and PC uptake in comparison to an empty vector (e.v.) control. All experiments were repeated at least two times with identical results.

For further functional analysis of lipid uptake at the plasma membrane, the flippase-deficient *dnf1Δdnf2Δdrs2Δ* strain expressing *APT1-4* constructs with or without *CDC50* or carrying an empty vector as negative control were incubated with fluorescent NBD-labeled lipids. Following the removal of surface-exposed probe, the remaining fluorescence was assayed by flow cytometry and the NBD-fluorescence of each transformant was plotted relative to the empty vector (Figure 6). For each given NBD-lipid, increased uptake would result in a higher fluorescence signal and a right shift of the cell population compared to the empty vector control. Based on this, we were able to reproduce the flippase activity of Apt1p with its broad substrate specificity for all tested glycerophospholipids (PS, PE, PC, PG) and all ceramides except lactosyl ceramide (GlcCer, GalCer, SM) (Figure 6A,B) [19]. In contrast, Apt2p shows specific, Cdc50p-dependent transport of PE and PC (Figure 6B,C). No flippase activity at the plasma membrane could be detected for Apt3p or Apt4p for the tested lipids (Figure 6A,B).

## 4. Discussion

This study provides the first biochemical analysis regarding substrate preference and subunit requirements among the *C. neoformans* P4-ATPases Apt2-4p based on heterologous expression in the flippase-deficient *S. cerevisiae* strain *dnf1Δdnf2Δdrs2Δ*. This expression system was used previously in our laboratory to characterize Apt1p as a lipid flippase of broad substrate specificity [19]. Here, we studied Apt2-4p in comparison to Apt1p. Western blotting confirmed the successful expression of Apt1-4 proteins together with the Cdc50 protein, with Apt2p showing lower relative expression levels. Sucrose density gradient fractionation showed that Apt1p and Apt3p localize to the plasma membrane, when co-expressed with Cdc50p. A minor fraction of Apt4p localizes to the plasma membrane independently of Cdc50p, while Apt2p appears to be restricted to inner membranes in both cases. However, due to low expression levels, it cannot be ruled out that Apt2p reaches the plasma membrane in amounts below the detection limit. In fact, since cells expressing *APT2* displayed uptake of exogenously applied NBD-lipids, at least a portion of the protein must reach the plasma membrane. Given that heterologous overexpression can lead to protein mislocalization [42], the localization of Apt2-4p in Cryptococcus remains to be confirmed.

**Figure 7:**
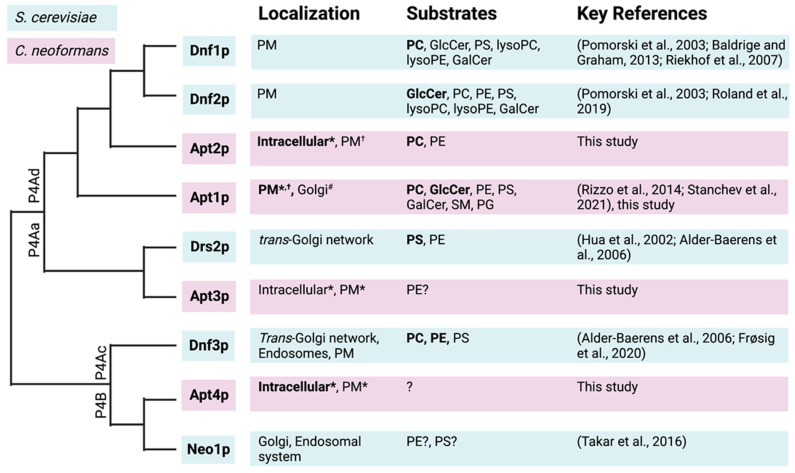
Phylogenetic comparisons of P4-ATPases from *S. cerevisiae* and *C. neoformans*. The phylogenic tree was generated using MUSCLE and sequences from Uniprot. Localization and substrate specificities are indicated based on key references. Subclade divisions within the P4A clade are as described in [51]. For accession numbers, see Materials and Methods. References: [15,19,25,26,52,53,54,55,56,57]. PM, plasma membrane; PE, phosphatidylethanolamine; PC, phosphatidylcholine; PS, phosphatidylserine; PG, phosphatidylglycerol; GlcCer, glucosyl ceramide; GalCer, galactosyl ceramide; SM, sphingomyelin. * based on subcellular fractionation; ^†^ based on fluorescence microscopy and lipid uptake studies; ^#^ putative.

Direct interaction with Cdc50p was confirmed for Apt1p and Apt3p after detergent solubilization and co-immunoprecipitation, but these assays were unsuccessful for Apt2p and Apt4p due to failed solubilization, which is probably a consequence of the heterologous expression system employed. However, recent structural studies on yeast and mammalian P4A-ATPases demonstrated that a tight interaction between the transporter and its β-subunit is necessary for lipid coordination and translocation [43,44,45,46,47,48]. In our work, Apt2p was found to require Cdc50p for flippase activity, both in functional complementation and lipid uptake assays (Figure 4 and Figure 6), which indicates that the proteins are directly interacting to form an active complex. Phylogenetic analysis revealed that Apt2p and Apt3p both belong to the P4A-ATPase subclade, which is in agreement with our finding that they both require a β-subunit for functionality (Figure 7; [19]). The requirement of Apt1-3p for Cdc50p explains the complex phenotypes observed for *C. neoformans* cells deleted in *CDC50*, which include PS exposure to the outer membrane leaflet, reduced virulence, and higher drug sensitivity [9,17].

Apt2p shares a high sequence similarity with Dnf1/2p but displays a narrower substrate specificity, limited to PC and PE (Figure 7). In contrast to mammals, where PC is evenly distributed among both leaflets of the plasma membrane or accumulated in the outer leaflet, yeast appears to maintain PC almost completely in the cytosolic leaflet [49,50]. Thus, Apt2p in complex with Cdc50p might help restrict both PE and PC to the cytoplasmic leaflet of the Cryptococcus plasma membrane, thus contributing to transbilayer lipid asymmetry. While the main localization of Apt2p, at least in Saccharomyces, seemed to be in endomembranes, Neo1p has been shown to contribute to the PS and PE asymmetry of the plasma membrane from an internal location corresponding to Golgi/endosomal compartments [25]. Apt-dependent clearance of PC and other phospholipids from the cell surface would lead to an enrichment of sphingolipids in the exoplasmic leaflet. As sphingolipids have saturated acyl chains and therefore pack at a higher density than glycerophospholipids, their enrichment in the exoplasmic leaflet of the plasma membrane would support its barrier function.

**Figure 8:**
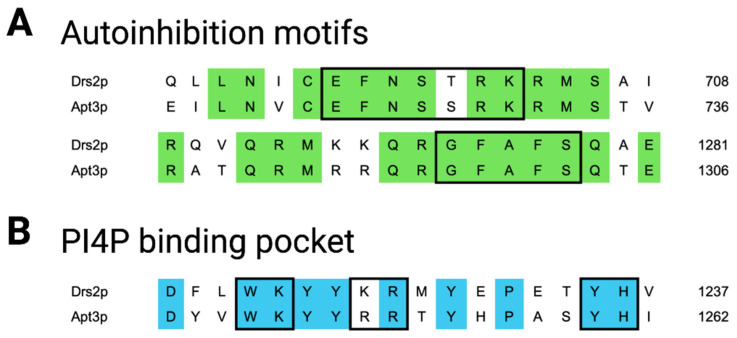
Conserved motifs from Drs2p in Apt3p. Sequence alignment of *S. cerevisiae* Drs2p and *C. neoformans* Apt3p via MUSCLE revealed the conservation of the previously identified Drs2p C-terminal autoinhibition site together with its binding site within the large cytosolic loop (**A**), as well as the PI4P binding site (**B**). Conserved amino acids are shown in green/blue, and the amino acids identified as important for the motif are highlighted in a box.

Apt3p and Drs2p share 53.9% sequence identity and our results show that both proteins also share functional similarity. First, the cold-sensitivity phenotype of the *dnf1Δdnf2Δdrs2Δ* strain, related to the *drs2* deletion [26], was rescued by *APT3/CDC50* co-expression. Second, the *APT3/CDC50*-complemented cells displayed higher fitness on galactose and miltefosine media as compared to the parental strain. Third, in line with the reported PE-transport activity for Drs2p [58], a low but significant resistance to duramycin was observed for *APT3/CDC50*-complemented cells. Given that Drs2p serves a critical role in membrane trafficking events from the trans-Golgi, Apt3p could serve a similar function in Cryptococcus. In agreement with this notion, deletion of *APT3* in *C. neoformans* renders these cells sensitive to the secretory pathway inhibitor BFA [17]. Previous work identified amino acid motifs at the C-terminal end of Drs2p that are required for its regulation [12,13,46,59,60,61]. Among these, a GFAFS sequence at the C-terminus interacts with an EFNSTRK motif located in the cytosolic loop between TM4 and TM5, overlapping the nucleotide-binding domain and inhibiting ATPase activity. This autoinhibition is partially released by binding of phosphatidylinositol-4 phosphate (PI4P) to a highly positively charged pocket upstream of the GFAFS domain in the interface with the membrane. All motifs required for Drs2p autoinhibition, and especially the core residues, are extremely well-conserved in Apt3p (Figure 8), suggesting that both proteins are not only functional homologs, but also similarly regulated.

Apt4p belongs to the subclass of P4B-ATPases, which also includes *S. cerevisiae* Neo1p (Figure 7) and ATP9A and ATP9B in mammals [62]. This subclass does not appear to use a β-subunit, suggesting that Apt4p might be able to function independently of Cdc50p [25,63]. Whether this holds true remains to be investigated. We observed a shift in the subcellular localization upon co-expression of Apt4p with Cdc50p but did not observe complementation of the *neo1-1^ts^* mutant, neither in the presence or in absence of Cdc50p. Likewise, the physiological role of Apt4p remains to be elucidated. In yeast and mammals, members of the P4B-ATPase subclass localize primarily to the Golgi and the endosomal system [25,62] and serve a role in the recycling of endosomes [64].

Taken together, our results provide a first insight into the biochemical characteristics of the P4-ATPases Apt2p and Apt3p from *C. neoformans*. The Saccharomyces expression strategy presented here provides a platform for future purification and reconstitution to enable further detailed molecular analysis. The recent design of an antifungal peptide that binds the Cdc50p extracellular loop demonstrates that P4-ATPases are a viable pharmacological target to bypass drug resistance in Cryptococcus [24]. In this context, the heterologous expression system presented here can be a powerful tool for more comprehensive inhibitor screenings.

## Data Availability

All the data are within the article and Supplementary Materials. All the data are to be shared upon request (Thomas Günther Pomorski, thomas.guenther-pomorski@rub.de).

## Supplementary Materials

The following supporting information can be downloaded at: https://www.mdpi.com/article/10.3390/jof9020202/s1, Figure S1: Topology prediction of Apt1-4p and Cdc50p from *Cryptococcus neoformans*; Figure S2: Intracellular localization of GFP-tagged Apt proteins upon heterologous expression in the P4-ATPase-deficient *S. cerevisiae* strain *dnf1Δdnf2Δdrs2Δ*. Table S1: Primer sets used for cloning; Table S2: Yeast expression plasmids used in this study.
