# Supplementary material for: Functional Analysis of the P-Type ATPases Apt2-4 from Cryptococcus neoformans by Heterologous Expression in Saccharomyces cerevisiae
Source: J Fungi (Basel). 2023 Feb 4;9(2):202. doi: 10.3390/jof9020202 (PMC9966271; doi:10.3390/jof9020202)
Supplement: Supplementary file 1 [file jof-09-00202-s001.zip › 3.jof-2156328-supplementary.pdf]

Functional analysis of the P-type ATPases Apt2-4 from *Cryptococcus neoformans* by heterologous expression in *Saccharomyces cerevisiae*

Sarina Veit [1], Sabine Laerbusch [1], Rosa L. López-Marqués [2,*], Thomas Günther Pomorski [1,2,*]

Supplementary Information

This file includes all supplementary information for the manuscript:

- Tables S1 and S2
- Figures S1 and S2

Supplementary Table S1: Primer sets used for cloning.

Set	Name	Sequence
#2-Nt	APT2- Frag1.FOR	5'-AAAAAACCCCGGATCCATGACTGCCTCTACTTATCAATCT-CATGG-3'
	APT2- Frag1.REV	5-'CTGAATTCGAATTCCTCCTCCGATAAATTTCTATACGC-3'
#2-Ct	APT2- Frag2.FOR	5'-GGAATTCGAATTCAGCGATTGGTCAAAAAAATATGACAC-3'
	APT2-Frag2.REV	5'-CCAGAACCTATAGCATATCCTGCACCTTCCCATTCG-3'
#2-myc	APT2spacer-myc-for	5'-TGCTATAGGTTCTGGTTCTGGTTCTGGTTCTGA-3'
	APT2-4-myc-rev	5'-GTCGTATTACGGATCCTTACTCGAGGTCTTCTTCGGAAA-TCAACTTCTG-3'
#2-GFP	APT2-GFP-Frag3.FOR	5'-TGCTATAGGTTCTGGTTCTGGTTCTGGTTCTA-3'
	APT2-4-GFP-Frag3.REV	5'-GTCGTATTACGGATCCTTACTTGTACAGC-3'
#3	APT3-Frag1.FOR	5'-AAAAAACCCCGGATCCATGCCTGCGCAACAGC-3'
	APT3-Frag1.REV	5'-CCAGAACCATACCCTGAAGGCCTCGC-3'
#3-myc	APT3spacer-myc-for	5'-AGGGTATGGTTCTGGTTCTGGTTCTGGTTCTGA-3'
	APT2-4-myc-rev	5'-GTCGTATTACGGATCCTTACTCGAGGTCTTCTTCGG-AAATCAACTTCTG-3'
#3-GFP	APT3-GFP-Frag2.FOR	5'-AGGGTATGGTTCTGGTTCTGGTTCTGGTTCTA-3'
	APT2-4-GFP-Frag3.REV	5'-GTCGTATTACGGATCCTTACTTGTACAGC-3'
#4	APT4-Frag1.FOR	5'-AAAAAACCCCGGATCCATGTCAACCTACTCGCGCACG-3'
	APT4-Frag1.REV	5'-CCAGAACCTATCCCAGCCACCTTCTTGTAAGCC-3'
#4-myc	APT4spacer-myc-for	5'- TGGGATAGGTTCTGGTTCTGGTTCTGGTTCTGA-3'
	APT2-4-myc-rev	5'-GTCGTATTACGGATCCTTACTCGAGGTCTTCTTCGGAAA-TCAACTTCTG-3'
#4-GFP	APT4-GFP-GFP-Frag2.FOR	5'-TGGGATAGGTTCTGGTTCTGGTTCTGGTTCTA-3′
	APT2-4-GFP-Frag3.REV	5'-GTCGTATTACGGATCCTTACTTGTACAGC-3'

Supplementary table S2: Yeast expression plasmids used in this study.

Name	Marker	Description	Source
pTP022	URA	pESC-URA vector (#217454)	Agilent Technologies (Santa Clara, CA, USA)
pTP772	URA	APT1-GFP CDC50-FLAG	(Stanchev et al., 2021)
pTP768	URA	APT1-GFP	(Stanchev et al., 2021)
pTP771	URA	APT1-myc CDC50-FLAG	(Stanchev et al., 2021)
pTP767	URA	APT1-myc	(Stanchev et al., 2021)
pTP808	URA	APT2-GFP CDC50-FLAG	This study
PTP724	URA	APT2-GFP	This study
pTP815	URA	APT2-myc CDC50-FLAG	This study
pTP723	URA	APT2-myc	This study
pTP809	URA	APT3-GFP CDC50-FLAG	This study
pTP726	URA	APT3-GFP	This study
pTP816	URA	APT3-myc CDC50-FLAG	This study
pTP725	URA	APT3-myc	This study
pTP810	URA	APT4-GFP CDC50-FLAG	This study
pTP819	URA	APT4-GFP	This study
pTP817	URA	APT4-myc CDC50-FLAG	This study
pTP818	URA	APT4-myc	This study

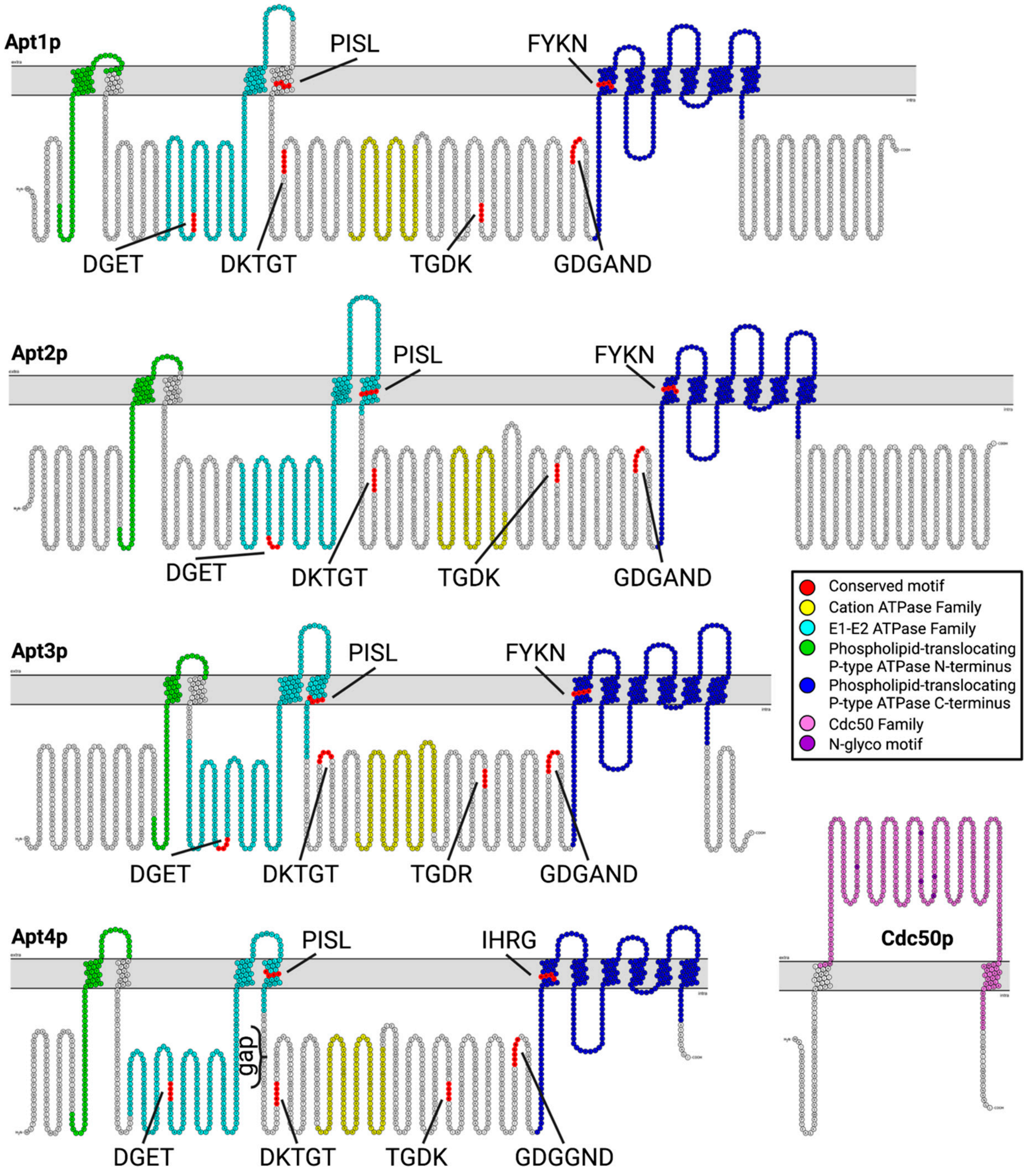

Figure S1: Topology prediction of Apt1-4p and Cdc50p from *Cryptococcus neoformans*. Transmembrane domains were predicted by sequence alignment with *S. cerevisiae* homolog and structure prediction with SWISS-MODEL. Protein family conserved domains were determined with Pfam and conserved P4-ATPase motifs are highlighted in red. Visualization done with Protter.

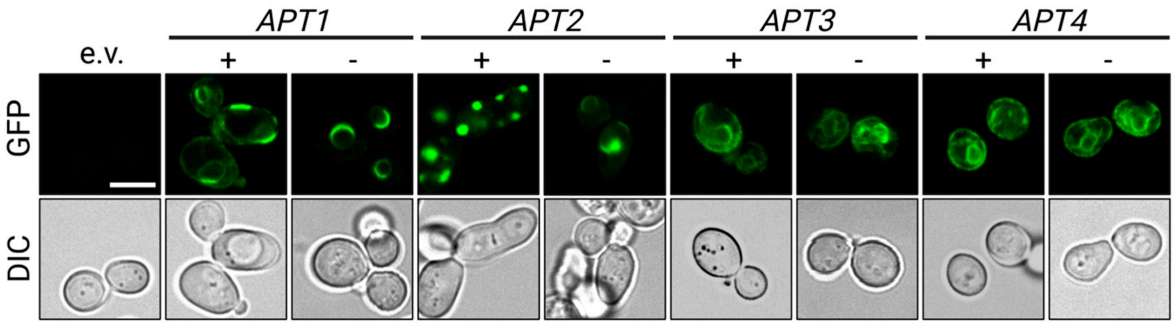

Figure S2: Intracellular localization of GFP-tagged Apt proteins upon heterologous expression in the P4-ATPase-deficient *S. cerevisiae* strain *dnf1Δdnf2Δdrs2Δ*. Cells transformed with an empty plasmid vector (e.v.) or with plasmids expressing the indicated GFP-tagged APT variants with (+) or without (-) *CDC50-FLAG* were grown in SD-U medium and induced in SG-U medium at 25°C for 24 h. Cells were analysed using differential interference contrast (DIC) microscopy and confocal fluorescence microscopy (GFP). Image acquisition were carried out on living cells using a spectral confocal laser scanning microscope (Leica TCS SP8) using a 63×/1.4 numerical-aperture (NA) oil objective. GFP was excited at 488 nm, and emission signals were recorded between 500 and 550 nm. Scale bar, 5 μm.
